# A Novel Hybrid Model for Predicting Blast-Induced Ground Vibration Based on k-Nearest Neighbors and Particle Swarm Optimization

**DOI:** 10.1038/s41598-019-50262-5

**Published:** 2019-09-27

**Authors:** Xuan-Nam Bui, Pirat Jaroonpattanapong, Hoang Nguyen, Quang-Hieu Tran, Nguyen Quoc Long

**Affiliations:** 1 Department of Surface Mining, Mining Faculty, Hanoi University of Mining and Geology, 18 Vien street, Duc Thang ward, Bac Tu Liem district, Hanoi, Vietnam; 2 Center for Mining, Electro-Mechanical research, Hanoi University of Mining and Geology, 18 Vien street, Duc Thang ward, Bac Tu Liem district, Hanoi, Vietnam; 3 Department of Mining and Petroleum, Chiang Mai University, 239 Huy Kaew rd., M., Chiang Mai, Thailand; 4 Institute of Research and Development, Duy Tan University, Da Nang 550000, Vietnam; 5 Department of Mine Surveying, Hanoi University of Mining and Geology, 18 Vien street, Duc Thang ward, Bac Tu Liem district, Hanoi, Vietnam

**Keywords:** Environmental impact, Natural hazards

## Abstract

In this scientific report, a new technique of artificial intelligence which is based on k-nearest neighbors (KNN) and particle swarm optimization (PSO), named as PSO-KNN, was developed and proposed for estimating blast-induced ground vibration (PPV). In the proposed PSO-KNN, the hyper-parameters of the KNN were searched and optimized by the PSO. Accordingly, three forms of kernel function of the KNN were used, Quartic (Q), Tri weight (T), and Cosine (C), which result in three models and abbreviated as PSO-KNN-Q, PSO-KNN-T, and PSO-KNN-C models. The valid of the proposed models was surveyed through comparing with those of benchmarks, random forest (RF), support vector regression (SVR), and an empirical technique. A total of 152 blasting events were recorded and analyzed for this aim. Herein, maximum explosive per blast delay (W) and the distance of PPV measurement (R), were used as the two input parameters for predicting PPV. RMSE, R^2^, and MAE were utilized as performance indicators for evaluating the models’ accuracy. The outcomes instruct that the PSO algorithm significantly improved the efficiency of the PSO-KNN-Q, PSO-KNN-T, and PSO-KNN-C models. Compared to the three benchmarks models (i.e., RF, SVR, and empirical), the PSO-KNN-T model (RMSE = 0.797, R^2^ = 0.977, and MAE = 0.385) performed better; therefore, it can be introduced as a powerful tool, which can be used in practical blasting for reducing unwanted elements induced by PPV in surface mines.

## Introduction

Blasting for rock fragmentation is known as one of the most impressive techniques in the fields of mining and civil engineering. However, it is estimated that only about 20% of the total explosive energy was used for rock fragmentation^1–4^. The remaining of explosive energy is wasted, which cause various undesirable effects to the environment, like, air over-pressure (AOp), flyrock, ground vibration, and back-break^5–7^. Of these effects, ground vibration, that is calculated using peak particle velocity (PPV), is utilized to be the most adverse parameter due to it can cause structural vibration, demolish structures, include instability of bench and slope, and affects the underground water^8–12^. Therefore, precise estimation of blast-produced PPV was needed to decrease its influence on our environment.

Until now, experimental and artificial intelligence (AI) commonly utilized for predicting blast-induced PPV^13^. The first one aims to establish empirical equations based on relationships between explosive charge per blasting delay (W) and the distance of PPV measurement (R)^14–23^. However, these empirical equations provide poor prediction performance in some cases e.g.^24–29^; therefore, the latter is considered.

Literature review shows that AI has proven its various efficient fields with promising performance, especially in advanced engineering as well as in mining and measurement^30–47,48^. In order to estimate blast-induced PPV, Khandelwal and Singh^10^ have successfully developed an artificial neural network (ANN) utilizing 154 blasting events at a surface coal mine in India with the conclusion that ANN is a powerful tool to estimate blast-induced PPV. Saadat, *et al*.^27^ also explored an ANN model to predict blast-induced PPV of an iron mine in Iran (Gol-E-Gohar) has been reported using 69 blasting events, even a proper result. Using other AI technique (i.e., classification and regression tree – CART), Khandelwal, *et al*.^49^ also successfully predicted PPV with high accuracy based on 51 datasets. Based on the advantages of the XGBoost model, Nguyen, *et al*.^50^ also investigated and predicted PPV with high performance using 136 datasets (i.e., RMSE = 1.742, R^2^ = 0.952). In another work, Nguyen, *et al*.^51^ optimized the Cubist models by a clustering technique (i.e., hierarchical K-means), for predicting PPV with high reliability. They concluded that the clustering technique can be considered as a robust technique in the classification of the dataset, as well as optimization of the Cubist models. In another work, Hasanipanah, *et al*.^52^ utilized the PSO algorithm to predict blast-caused PPV, where two forms, power (P) and linear (L) were used. An empirical technique, along with MLR analysis, are also used for comparing with those of the two PSO models. They reported that the PSO-P provides high prediction performance. Armaghani, *et al*.^53^ investigated an integration of PSO with ANN in order to estimate blast-induced PPV, namely PSO-ANN model. They utilized the algorithm of PSO for optimizing the network architecture of the ANN model. A series of empirical equations are additionally applied to estimate PPV and compare with those of the PSO-ANN model. Conclusion of their study is that the PSO-ANN model yielded an outstanding result. In another study, Armaghani, *et al*.^54^ used the ICA optimization to estimate blast-induced PPV utilizing 73 blasting events and also a suitable result was determined in their work. Based on the ICA, Hasanipanah, *et al*.^55^ also introduced a fuzzy system (FS) model for estimating the model of blast-induced PPV, i.e., FS-ICA. For performing comparisons, a variety of empirical models were also calculated in their study, which proved that the model of FS–ICA outperforms the other experimental approaches. By the use of another optimization algorithm (i.e., firefly algorithm-FFA), Shang, *et al*.^56^ developed a new technique to predict PPV using FFA-ANN model. Zhang, *et al*.^57^ also developed the PSO-XGBoost technique for the aim of PPV prediction with high performance. In addition, PSO-ANFIS and GA-ANFIS were also investigated by Yang, *et al*.^58^, for predicting PPV. Table 1 lists some studies concerning the prediction of blast-induced PPV using AI techniques.

**Table 1:** Some studies concerning the prediction of blast-induced PPV using AI techniques.

References	AI technique
Singh and Singh^8^	ANN
Khandelwal and Singh^61^	ANN
Khandelwal and Singh^9^	ANN
Iphar, *et al*.^107^	ANFIS
Khandelwal and Singh^10^	ANN
Khandelwal, *et al*.^11^	SVM
Monjezi, *et al*.^108^	ANN
Monjezi, *et al*.^24^	ANN
Khandelwal, *et al*.^62^	ANN
Ghasemi, *et al*.^25^	FL
Monjezi, *et al*.^26^	ANN
Saadat, *et al*.^27^	ANN
Armaghani, *et al*.^53^	PSO-ANN
Hasanipanah, *et al*.^109^	SVM
Dindarloo^110^	GA
Hajihassani, *et al*.^28^	ICA-ANN
Hajihassani, *et al*.^111^	PSO-ANN
Amiri, *et al*.^83^	ANN-KNN
Monjezi, *et al*.^112^	GEP
Hasanipanah, *et al*.^113^	CART
Hasanipanah, *et al*.^52^	PSO
Taheri, *et al*.^114^	ABC-ANN
Ragam and Nimaje^115^	GRNN
Armaghani, *et al*.^116^	ICA
Behzadafshar, *et al*.^117^	ICA
Sheykhi, *et al*.^118^	FCM-SVR
Arthur, *et al*.^119^	GP

Note: adaptive neuro-fuzzy inference apparatus (ANFIS); support vector machine (SVM); gene expression programming (GEP); fuzzy logic (FL); genetic algorithm (GA); classification and regression tree (CART); artificial bee colony algorithm (ABC); generalized regression neural network (GRNN); fuzzy C-means clustering (FCM); Gaussian process (GP).

We have found that optimization algorithms are becoming a powerful tool for estimating blast-induced PPV, notably the PSO algorithm. They play a considerable role in the case of enhancing the efficiency of models. However, it was only considered for ANN and XGBoost models. Nevertheless, new hybrid models are needed for knowledge and practical engineering to reduce the undesirable influences of blasting operations. In this work, we expanded the body of knowledge by proposed the PSO optimized *k*-nearest neighbors (KNN) and named as PSO-KNN for estimating blast-induced PPV. The RF, SVR, and empirical models were also considered and exploited to predict PPV based on the same dataset.

## Materials

**Figure 1:**
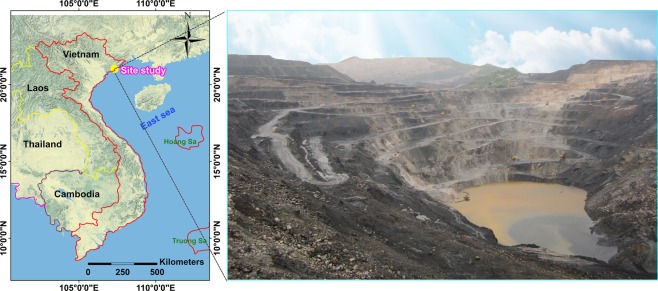
Location and landscape of the study site.

In this study, blasting operations were undertaken at the Deo Nai open-pit coal mine for rock fragmentation. The study site locates in the North of Vietnam, between latitudes 21°01′00″N and 21°20′00″N, and between longitudes 107°18′15″E and 107°19′20″E (Fig. 1). The Arcmap version 10.2 (Link: http://desktop.arcgis.com/en/arcmap/) was used to create the map in Fig. 1. The total area of this mine is ~6.0 km^2^ with exploitation reserve of 42.5 Mt, and fertility of 2.5 Mt/yr^59^.

The geological structure in the mine is very complicated. Many interleaved faults and folds divide the deposit into many different complex blocks. In this mine, the volume of the overburden is 20 to 30 million m^3^/yr. The main bulk of the overburden includes conglomerate, sandstone, siltstone, claystone, and coal clay. Therefore, in this mine, blasting is considered to be imperative for fragmenting rocks. ANFO explosive (ammonium nitrate–fuel oil) was used as the primary explosive in this mine with the hole diameter in the range of 150 to 250 mm. Note that, the non-electric delay blasting method^15,60^ is used in this mine in the case of rock breakage.

**Figure 2:**
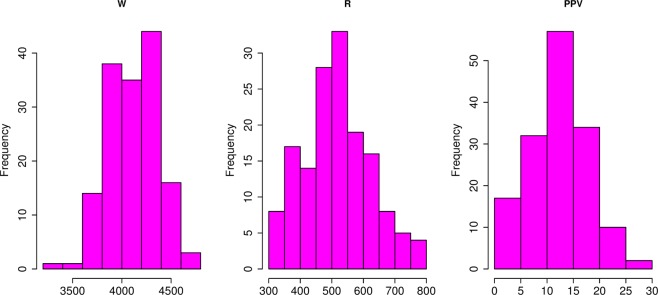
Histogram of the blast-induced ground vibration dataset.

As stated in the literature^61–63^, W and R have the most impacts on PPV, therefore, in this study, both of the W and R parameters are utilized as the primary input parameters for PPV estimation. The Blastmate III perspective (i.e., Instantel in Canada) is utilized for recording the PPV value. Note that the term R was defined by a handheld GPS where W was extracted from 152 blast patterns. Table 2 summaries the data taken in this work. Also, the histograms of each attribute are illustrated in Fig. 2.

**Table 2:** Properties of the data taken.

Properties	W	R	PPV
Min.	3200	308.2	2.050
1st Qu.	3952	448.0	8.545
Median	4135	513.0	12.435
Mean	4120	518.3	12.400
3rd Qu.	4295	574.1	15.980
Max.	4643	799.2	29.180

Note: W denotes the explosive charge per delay (in Kg); W indicates the monitoring distance (in m); PPV means the intensity of ground vibration (in mm/s).

## Methods

As mentioned above, the principal purpose of this work is to expand a novel hybrid model for estimating blast-induced PPV (i.e., called PSO-KNN model). Moreover, a practical technique and also two algorithms, (e.g., RF and SVR), are also utilized as benchmarks for estimating blast-induced PPV. However, the description of the RF and the SVR has been well documented, i.e., in^64–66^; therefore, the background of the RF and the SVR is not provided in this study.

### Empirical

From reviewing the lecture, we have shown that empirical equation of the U.S Bureau of Mines (USBM)^14^ is the most common technique where it has been widely applied to estimate PPV produced by blasting operations. Therefore, for the current research, it was implemented for predicting PPV and is demonstrated as:

1 $${\rm{PPV}}=\lambda {(\frac{{\rm{R}}}{\sqrt{{\rm{W}}}})}^{-\alpha }$$

W stands for the maximum explosive charge per delay (in Kg);R stands for the monitoring distance (m);where *λ* and *α* were the site parameters and were considered using the multivariate regression analysis.

### PSO algorithm

In the present work, the algorithm of PSO is utilized for optimizing the KNN model. In the regards of the PSO, more details have been presented in refs.^67–71^.

The PSO algorithm is one of the most efficient metaheuristic techniques proposed by Eberhart and Kennedy^72^. This method was adopted from the social animals/particles behavior, like a flock of birds in a swarm and can be used to predict optimization issues with every solution is illustrated as a particle. In order to determine the optimized solution, the algorithm of PSO considers the following steps^73^:

Step 1: Initialize population of particles as well as its related velocity. After that, predict the fitness of particles and discover the best location as local and global best.

Step 2**:** Each particle changes about quest zone with a particular velocity. For each iteration, global best and local best are calculated to assess the efficiency of the PSO-KNN models. Global best is considered as the best-gathered particle position, and the local best is regarded as the best solution in the prevalent iteration.

Step 3: Update the location of a particle**;** After predicting the velocity of particles, the positions of them change about quest zone with the calculated speed and for considered particles, the procedure can calculate and update the new velocity utilizing Eq. 2 as follow:

2 $$\begin{array}{c}{v}_{j}^{i+1}=w{v}_{j}^{(i)}+({c}_{1}\times {r}_{1}\times (local\,bes{t}_{j}-{x}_{j}^{(i)}))+({c}_{2}\times {r}_{2}\times (global\,bes{t}_{j}-{x}_{j}^{(i)})),{v}_{\min }\le {v}_{j}^{(i)}\\ \,\,\,\,\,\,\,\le {v}_{\max }\end{array}$$

where ${x}_{j}^{(i)}$ denotes the position of particle *j* at iteration *i;* ${v}_{j}^{(i)}$ means the particle velocity *j* for iteration *i*; *w* stands for the inertial weight coefficient; *i* stands for the number of iteration; *r*_1_ and *r*_2_ stand for the numbers in the interval [0,1].

- **The global best and the local best** can be updated when the new particle becomes to remove. The system was calculated and then updated the location, for each particle, using Eq. 3 as below:
  3 $${x}_{j}^{i+1}={x}_{j}^{(i)}+{v}_{j}^{(i+1)};\,j=1,2,\ldots ,n$$
- **Investigate the termination criteria**, when the principle of termination has been satisfied, change the global best as the proper and optimized solution for an issue.

### *k*-Nearest Neighbors (KNN)

The KNN is known as one of the non-parametric approaches in term of classification and regression issues^74^. The most critical parameters of the KNN algorithm are the number of nearest neighbors (*k*) and the distance metric (*d*). In regression problems, the parameter *k* specifies the number of neighbor observations that contribute to the output predictions (i.e., PPV. Instead of considering at the closest reference sample, the algorithm of KNN views at the *k* instances in the reference collect which is near to the unknown instance as well as performs a vote to make a decision^75,76^. More details in the case of the algorithm of KNN can be obtained in refs.^77–79^.

Review of previous works indicate that the KNN algorithms have been applied correctly in many fields^80–82^; however, it seems to be rarely considered for estimating blast-induced problems. Amiri, *et al*.^83^ Amiri et al. proposed the model ANN-KNN for the first time. ANN-KNN is composed of two component models of KNN and ANN. Each model predicts test samples, and the obtained outcome is a weighted combination of the findings. Firstly, they use K means clustering to partition the training sample within identical clusters. In order to predict a testing sample by KNN, the nearest teammate instance has been found utilizing the distance of city for the test sample. Then, the values of the factors of the closest train instance were considered to the test instance. For each cluster, besides KNN, an ANN can be trained to utilize the train instance of that cluster. Once the ANN models trained, they can be used to predict PPV on the same group of the testing dataset. However, optimization problems for the ANN model and the KNN approach for estimating blast-induced PPV in their work have not been implemented. The weights and ascending bias, as well as the hidden node of the ANN model, have been reviewed and calculated according to the experimental formulas. Likewise, the KNN model was also determined by the traditional method. Note that, in the present study, the training dataset is not divided by clustering algorithms. The KNN algorithm was applied to develop the KNN model on the whole of the training dataset with the hyper-parameters (*k*, *d*) put to use to tune the performance of KNN model. To define the most optimal values for the KNN model, the PSO method was included in the adjustment process of k and d of the KNN model.

### Proposing the PSO-KNN model

**Figure 3:**
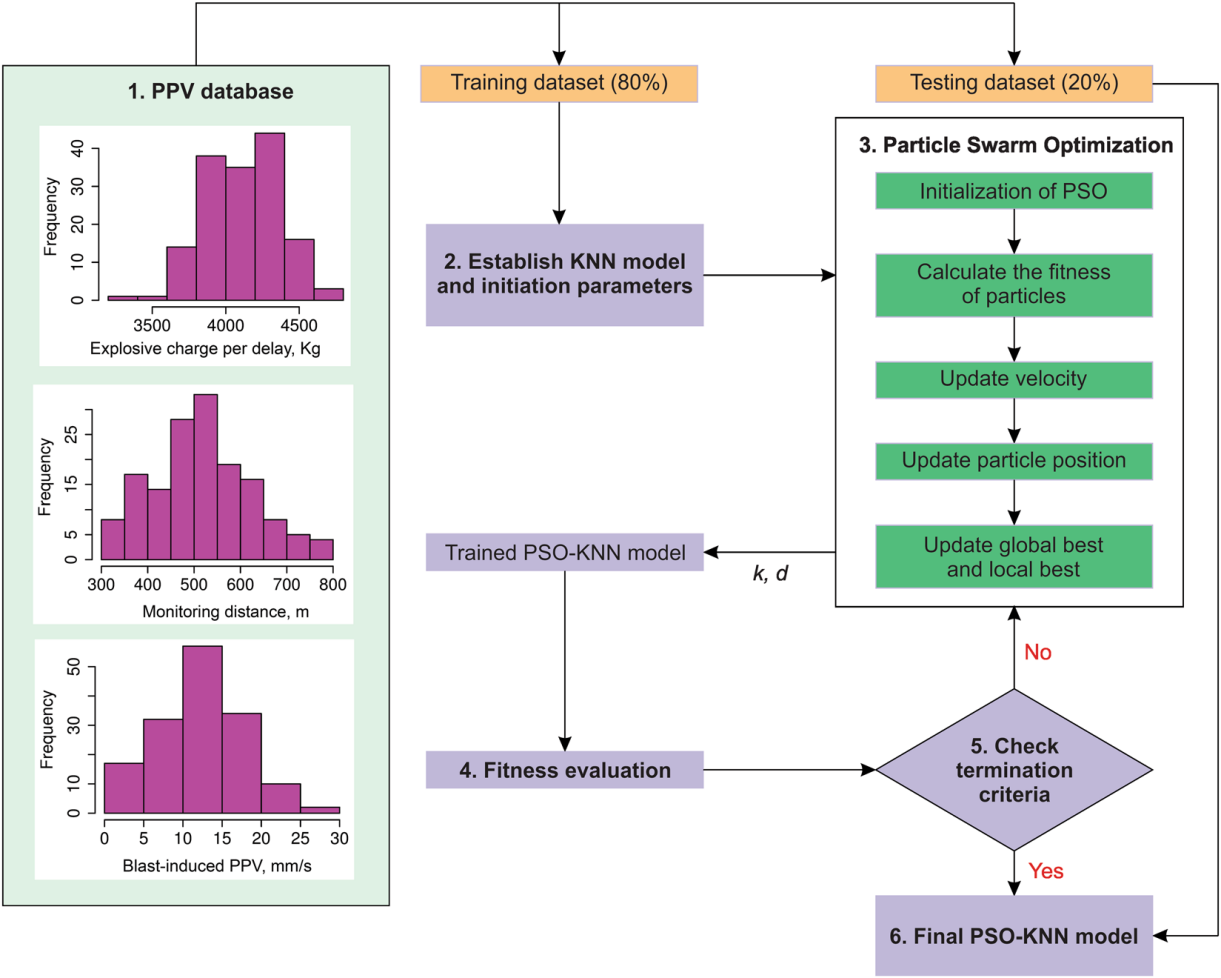
Scheme of a proposed PSO-KNN model for estimating blast-induced PPV.

In the present work, the KNN algorithm is the primary algorithm used to estimate blast-induced PPV. The two main hyper-parameters of the KNN model, including *k* and *d*, are utilized to adjust the efficiency of the algorithm. For determining the optimal values of *k* and *d*, the PSO algorithm was adopted. As shown in Fig. 3, the particles in PSO performed a global search procedure for the best *k* and *d* values of the KNN model, called PSO-KNN model. The expanding of the PSO-KNN algorithm has been accomplished through four steps as:

- Step 1: Making the PPV data and preparing the training and testing databases.

In this step, 152 blasting events were divided into two phases by randomly; 124 blasting activities in the first phase (~ about 80 percent of the whole dataset) are used for the training process to expand the PSO-KNN models. The rest 28 blasting events (~20%) in the second phase were used to check the efficiency of the constructed approaches.

- Step 2: Configuration of the KNN model.

As a criterion, the KNN model is considered as the dominant model to predict PPV in the present work. It is noted that three shapes of kernel function were applied for KNN, including quartic (Q), triweight (T), and cosine (C). These functions are described as following^74,84^:

4 $${\rm{Quartic}}:K(u)=\frac{15}{16}{(1-{u}^{2})}^{2}$$

5 $${\rm{Triweight}}:K(u)=\frac{35}{32}{(1-{u}^{2})}^{3}$$

6 $${\rm{Cosine}}:K(u)=\frac{\pi }{4}{\rm{c}}{\rm{o}}{\rm{s}}(\frac{\pi }{2}u)$$

where K is a function that can be integrated with non-negative real values. The primary purpose of using these kernel functions in the present study is to map the data to a higher dimension with the linear relationship. It makes regression of PPV values more accurate in modeling. More details of kernel functions for the KNN model can be found at the following references^74,84–87^.

- Step 3: Optimization of KNN, evaluation of fitness, and check termination criteria.

This step aimed to find an optimal KNN model with the lowest amount of a fitness function by searching the best amounts for the hyper-factors of KNN (*k*, *d*) using PSO algorithm. To perform the most appropriate KNN approaches according to the PSO algorithm, RMSE is computed as a fitness function as described in Eq. 7. The flowchart of the suggested PSO-KNN algorithm for estimating blast-induced PPV was illustrated in Fig. 3.

- Step 4: Final PPV predictive model.

After that, the optimization method by the algorithm of PSO is completed, the best hyper-parameters of the KNN model were derived and used to build the final PPV predictive methods. The goodness of the approaches was evaluated via the training dataset and performance statistical indexes like MAE, RMSE, and R^2^. The error distribution is provided by RMSE^88,89^ illustrating the idea of how proper an approach has adjusted the information via R^2^. In an optimal model, the RMSE, and MAE could be equal to zero whenever the R^2^ could be equal to 1. The performance indicators are computed as:

7 $${\rm{RMSE}}=\sqrt{\frac{1}{n}\mathop{\sum }\limits_{i=1}^{n}{({y}_{PPVi}-{\hat{y}}_{PPVi})}^{2}}$$

8 $${{\rm{R}}}^{{\rm{2}}}=1-\frac{\sum _{i}({y}_{PPVi}-{\hat{y}}_{PPVi}{)}^{2}}{\sum _{i}{({y}_{PPVi}-\overline{y})}^{2}}$$

9 $${\rm{MAE}}=\frac{1}{n}\mathop{\sum }\limits_{i=1}^{n}|{y}_{PPVi}-{\hat{y}}_{PPVi}|$$

*n* stands for a total number of observations; ${y}_{PPVi}$ is the measured PPV,${\hat{y}}_{PPVi}$ is predicted PPV, and $\overline{y}$ is the mean of ${y}_{PPVi}$.

## Establishing the Predictive Models

In order to develop PPV predictive models in this work, the database, including 152 blasting events, was split into two parts. According to Nick^90^, the most usually utilized train/test ratio was 80:20, which was a proper starting ratio based on Swingler^91,92^; hence, 80% of the total information (around 124 events of blasting) is used as the training database for the first section; the remaining amount that consists of 28 blasting events was recognized as the testing database in the second section.

### Empirical model

For the empirical model, *λ* and *α* are the site parameters and are found using an analysis of multivariate regression. In the present work, the SPSS method (version 16.0) is employed to specify *λ* and *α* according to 124 blasting events of the training database. We found that *λ* = 0.051 and *α* = −2.596 are the optimized amounts for the site parameters. In this work, the empirical equation USBM is illustrated as below:

10 $${\rm{PPV}}=0.051{(\frac{{\rm{R}}}{\sqrt{{\rm{W}}}})}^{2.596}$$

### RF model

**Figure 4:**
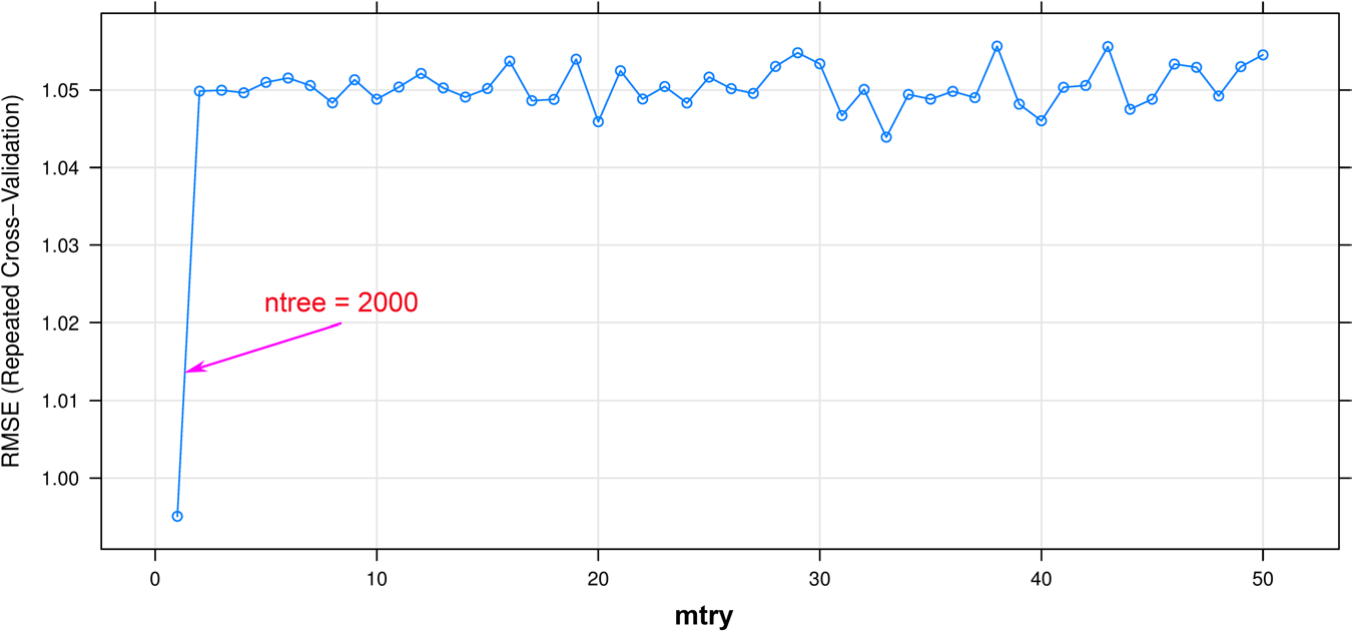
Efficiency of the RF algorithm on the training database.

RF is considered as the best decision tree methods suggested by Breiman^93^. It may predict both classification and regression issues, i.e., predict PPV. For this aim, the number of the tree (*ntree*) and randomly selected predictor (*mtry*) are the main hyper-parameters involving to adjust the quality of the RF approach. Theoretically, *ntree* must be large enough to avouch the wealth and objectivity of the forest^94^. Each decision tree in the forest acts as a voter. Therefore, *ntree* was set equal to 2000. To introduce the optimized value of the *mtry* parameter, the grid search approach^95^ was applied with *mtry* in the range of 1 to 50; The 10-fold cross-validation resampling method^96^ is used to avoid over-fitting for RF model. As a result, *ntree* = 2000 and *mtry* = 1 were the best for the RF model (Fig. 4). Its performance was evaluated through RMSE, MAE, and R^2^ on the training dataset.

### SVR model

**Figure 5:**
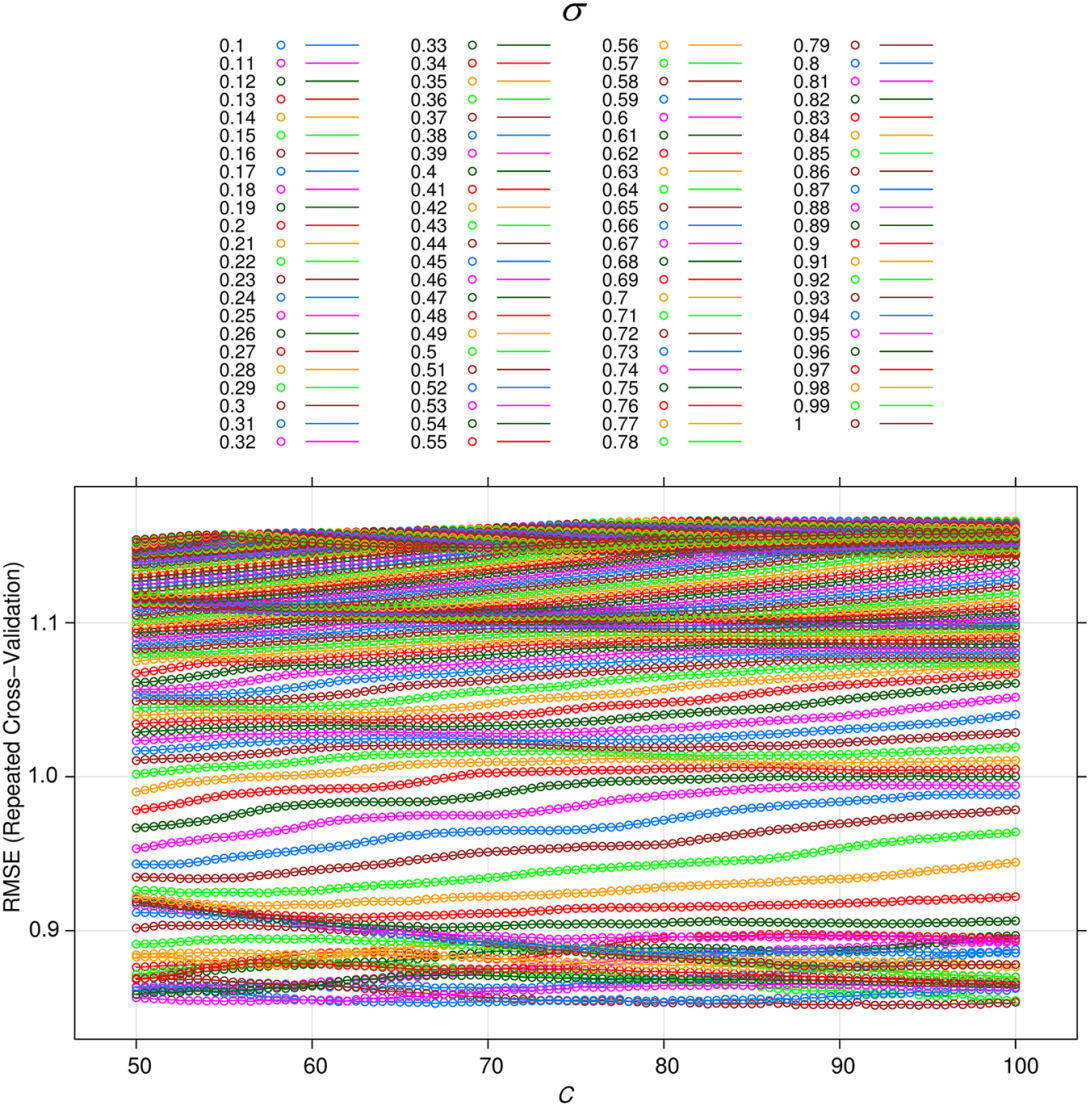
Efficiency of the SVR method on the training dataset.

For SVR model, the regression problem of PPV was employed through a kernel function. Many previous scientists recommended that the radial basis function (RBF) should be applied in SVR for regression problems with more high accuracy^97–99^. Therefore, the function of the RBF kernel is chosen selected for SVR model with *σ* and *C* were the RBF’s parameters. The 10-fold cross-validation resampling method is implemented for the SVR model to avoid over-fitting. In the case of expansion of the SVR method, a grid search method for *σ* and *C* was established to discover the most proper amounts of the SVR. In this regard, *σ* was set in the range of 0.1 to around 1; *C* was adjusted in the range of 50 to 100. Eventually, an optimized SVR method is determined with *σ* = 0.16 and *C* = 94.5. Figure 5 indicates the performance of the SVR model for predicting PPV on the training dataset.

### PSO-KNN model

In this part, the development of the PSO-KNN method for estimating PPV was presented in detail. As stated earlier, this model is developed via a combination of KNN and PSO algorithms, as shown in Fig. 3. The training dataset for the proposed PSO-KNN method is identical with those utilized in the empirical, RF, and SVR models. According to the training dataset, as the first step, a primary KNN model is produced. Then, as the next step, the hyper-factors of the KNN method are improved using the algorithm of PSO. This part aimed to discover a PPV predictive method with the lowest RMSE by finding the best amounts for the hyperparameters by the algorithm of PSO. In this algorithm, maximum particle’s velocity (*V*_*max*_), maximum iteration number (*m*_*i*_), the population number (*p*), individual and group cognitive (*ϕ*_1_, *ϕ*_2_), as well as inertia weight (*w*), are the factors utilized for the optimization approach. The sample size should be large enough to ensure the population diversity^100,101^. Hence, a trial-and-error method is selected, and 50 individuals were the best for the immediate work area (*p* = 50). For the process of terminating the optimization, *m*_*i*_ is adjusted equally to 500^102^ for checking the particle positions fitness by utilizing the RMSE metric (Eq. 7). For ensuring the balance among global detection and also local search, *w* is adjusted equal to around 0.9^103^. Based on previous works, Kennedy^104^ and Clerc and Kennedy^105^, *ϕ*_1_ can be identical to *ϕ*_2_ and *ϕ*_1_ + *ϕ*_2_ lie in the range of 0 to 4. Hence, in the present work, *ϕ*_1_ = *ϕ*_2_ = 1.5. In order to ensure convergence along with preventing explosion^106^, *V*_*max*_ is adjusted equal to 2.

**Figure 6:**
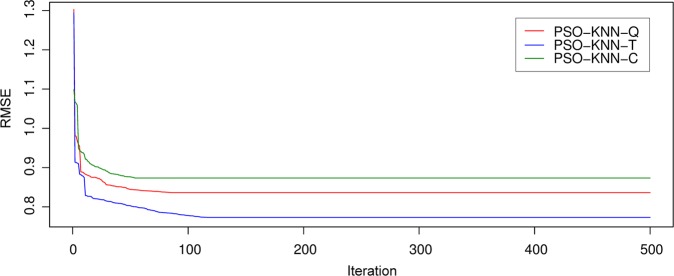
Efficiency of the PSO-KNN models in the process of optimization.

Once the PSO’s factors are adjusted, the compatibility of particle locations is calculated via the RMSE function. For any definition in the process of optimization, considered particles jump in a constrained checking zone and exchange their experiment to discover the best location (i.e., lowest RMSE); 500 iterations were used to determine the best factors of the suggested PSO-KNN model according to the best position (i.e., lowest RMSE) of the swarm of whole repeats. Note that, three forms of the kernel function (Q, T, C) were applied for the PSO-KNN model as described in the previous section. Figure 6 indicates the efficiency of the optimization approach for the PSO-KNN algorithms. Note that, the best amounts of the hyper-factors obtained for the PSO-KNN models (i.e., after the process of optimization) were determined in Table 3.

**Table 3:** The hyper-parameters of PSO-KNN models.

Model	Optimization values of the hyper-parameters	
	*k*	*d*
PSO-KNN-Q	20	0.493
PSO-KNN-T	10	0.503
PSO-KNN-C	16	0.499

## Results and Discussions

In the present section, the outcomes of the PPV predictive algorithms were highlighted. The efficiency indexes of the empirical, the RF, the SVR, and the three PSO-KNN models were evaluated based on RMSE, R^2^, and MAE, as illustrated in Table 4. The testing database is utilized as the unseen information to check the quality of the expanded models.

**Table 4:** Efficiency indexes of the PPV predictive approaches in this work.

Model	Training dataset			Testing dataset		
	RMSE	R^2^	MAE	RMSE	R^2^	MAE
Empirical	2.525	0.822	1.306	3.615	0.579	1.727
RF	0.995	0.966	0.508	1.126	0.952	0.499
SVR	0.852	0.973	0.574	1.175	0.944	0.634
PSO-KNN-Q	0.836	0.977	0.417	0.982	0.964	0.454
**PSO-KNN-T**	**0.773**	**0.982**	**0.403**	**0.797**	**0.977**	**0.385**
PSO-KNN-C	0.873	0.975	0.430	1.014	0.960	0.455

Note: the best model was shown in bold type.

**Figure 7:**
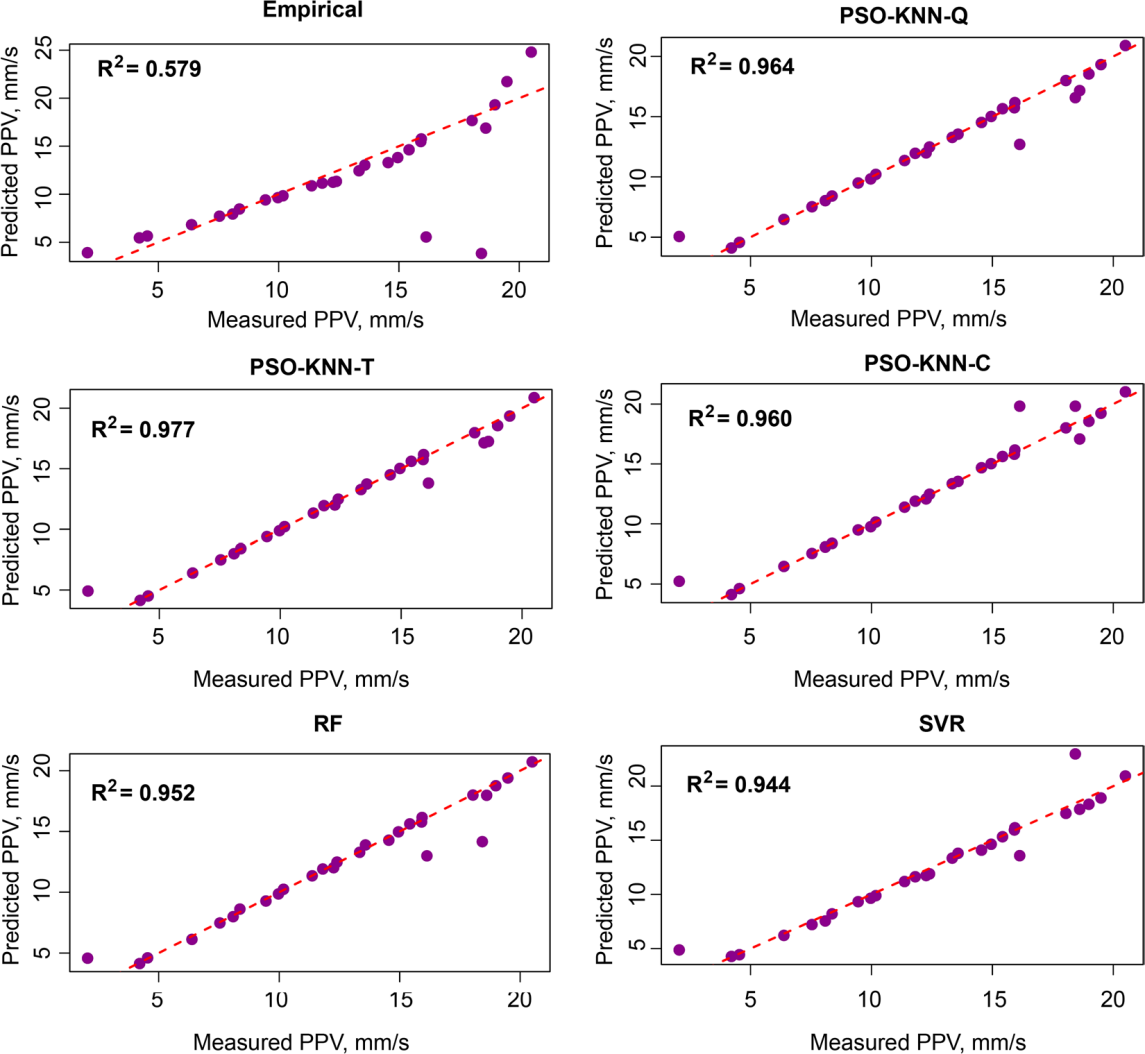
Measured versus predicted values of the models.

**Figure 8:**
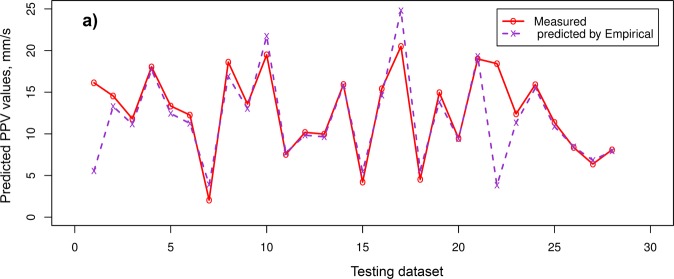
Comparison among exact and estimated amount using the empirical model.

**Figure 9:**
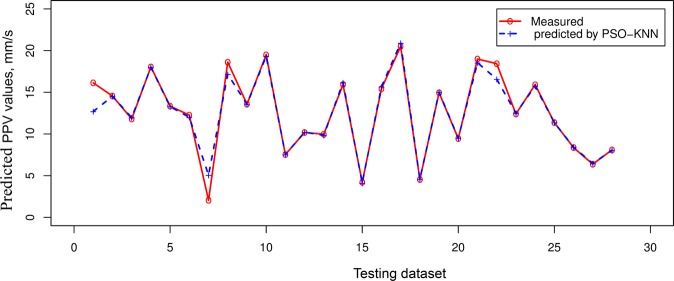
Comparison among exact and estimated amount using the PSO-KNN-T model.

**Figure 10:**
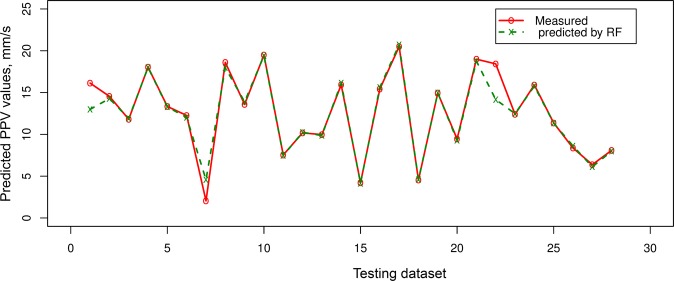
Comparison among exact and estimated amount using the RF model.

**Figure 11:**
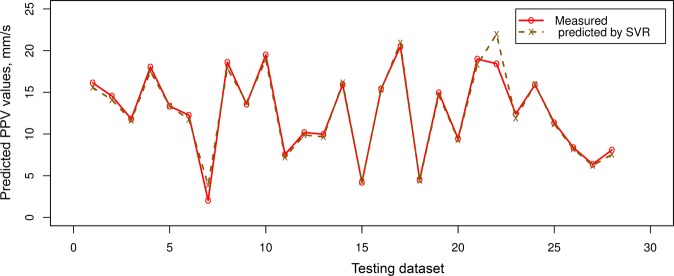
Comparison among exact and estimated amount using the SVR model.

Table 4 indicated that the PSO-KNN models properly performed compared to the empirical, RF, and SVR models in estimating PPV. On the training dataset, the PSO-KNN models obtained robust performance with the RMSE in the range of 0.773 to 0.873; R^2^ in the range of 0.975 to 0.982; MAE in the field of 0.403 to 0.430. The benchmark models (RF and SVR) were additionally performed quite suitable in this work. But their efficiency was poorer than the PSO-KNN models with an RMSE in the range of 0.852 to 0.995, R^2^ in the field of 0.966 to 0.973, and MAE in the range of 0.508 to 0.574. In contrast, the empirical model yielded the poorest performance (RMSE = 2.525, R^2^ = 0.822, and MAE = 1.306). Observing the efficiency of the models on the testing dataset, it may be observed that the PSO-KNN algorithms were also outperformed over the other models (RMSE = 0.797 to 1.014; R^2^ = 0.960 to 0.977; MAE i = 0.385 to 0.455). Remarkable, the PSO-KNN model with the triweight kernel function (PSO-KNN-T) yielded the most accuracy among the proposed PSO-KNN models (i.e., RMSE = 0.797, R^2^ = 0.977, and MAE = 0.385). Next are the PSO-KNN-Q, PSO-KNN-C, RF and SVR models with RNSE in the range of 0.982 to 1.175; R^2^ in the range of 0.944 to 0.964; MAE in the range of 0.454 to 0.634. In contrast, the empirical obtained the poorest performance on the testing dataset (i.e., RMSE = 3.615, R^2^ = 0.579, and MAE = 1.727). Based on the results in Table 4, all the models are well generalized, especially the PSO-KNN model with triweight kernel function (i.e., PSO-KNN-T) is an outstanding model in term of RMSE, R^2^, and MAE. Therefore, it was selected as the most appropriate model for estimating PPV produced by bench blasting. Figure 7 shows the efficiency of the models on the testing database. Also, the precision of the expanded models is even compared in Figs 8–11.

## Conclusions

Blasting is known as one of the most appropriate and cheapest approaches for the fragmentation of hard-rocks in the case of open-pit mines. Nevertheless, its improper impacts on the surrounding environment, particularly ground vibration (PPV), are unavoidable. Hence, precise blast-induced PPV estimations are essential for decreasing the effects on our environment. The present work proposed a new hybrid technique for estimating PPV according to the KNN and PSO algorithms with high accuracy, namely PSO-KNN. According to the outcomes of this work, authors obtain some results as follows:

- Blast-induced PPV is a usual involved and non-linear issue that is hard to investigate and estimate. High accuracy of the proposed PSO-KNN model in this study indicating that AI techniques are reasonable solutions, which solve this problem better than the empirical method.
- The PSO algorithm is a suitable optimization tool for estimating purposes of blast-induced PPV. It has a dramatic role in enhancing the precision of the KNN approach, according to RMSE, R^2^, and MAE, as illustrated in Table 4. However, the integration of PSO and KNN algorithms are often complexity when setting the parameters.
- The proposed PSO-KNN model (PSO-KNN-T) is a superior approach in estimating PPV induced by bench blasting; therefore, it is an alternative tool that should be considered for other areas in predicting PPV, as well as the other blasting problems in practical engineering.
- This research only considered two parameters of W and R for establishing the blast-induced PPV modes. Therefore, the performance of these models can be enhanced if the other parameters related to the blast pattern and properties of rock mass are to be considered.

## Data Availability

All data generated or analyzed during the current study are included.
